# Hematopoietic System under Physiological Conditions and Following Hematopoietic Reconstitution or Stress

**DOI:** 10.3390/ijms24108983

**Published:** 2023-05-19

**Authors:** Maria Kalashnikova, Alexander Belyavsky

**Affiliations:** 1 Engelhardt Institute of Molecular Biology, Russian Academy of Sciences, Vavilova 32, 119991 Moscow, Russia; alopexmary@gmail.com; 2 Institute of Higher Nervous Activity and Neurophysiology, Russian Academy of Sciences, Butlerova 5A, 117485 Moscow, Russia

The hematopoietic system performs the most vital functions in the human body, integrating the work of various organs while producing enormous numbers of mature cells daily. Hematopoiesis is primarily driven by hematopoietic stem and progenitor cells (HSPSCs) which, in performing this function, must carefully balance their decisions of self-renewal and differentiation while coordinating them with the state of the organism and its emerging needs—in particular, during various environmental stresses and challenges such as microbial infections. Experimental evidence of the last years, using the most advanced molecular, genetic and cellular techniques, reveals an unexpected complexity of both the HSPC compartment and the bone marrow (BM) niche where HSPCs reside. Intensive ongoing efforts worldwide concentrate on elucidating, in full detail, the intricacies of HSPC biology, as well their existence within the BM niche and complex HSPC niche interactions. This Special Issue was launched to increase the awareness of biomedical specialists in these important topics. It includes two experimental [1,2] and four review articles [3,4,5,6] reporting the latest advances in this prominent field of biomedicine.

The work of Garg et al. [1] reports new findings concerning the important issue of protection against ionizing radiation. The hematopoietic system is arguably the system most susceptible to radiation injury in the human body. Ionizing radiation elicits acute radiation syndrome (ARS), resulting in damage to actively proliferating stem/progenitor cells and ensuing pancytopenia, as well as poor recovery and delayed immune reconstitution [7,8]. Currently, only the radiomitigators can be used to combat ARS [9,10], while no radioprotectors have yet been approved by the FDA. Tocotrienols—and in particular, gamma tocotrienol—are potent antioxidants and free radical scavengers; thus, they are promising candidates to be used as radioprotectors [11]. The study published in this Special Issue is the first work to address the state of major immune cell populations in BM after total body radiation in non-human primates. The results obtained by Garg et al. demonstrate that pre-treatment of irradiated animals with gamma tocotrienol mitigates radiation-induced injury to hematopoietic cells. The study also showed that gamma tocotrienol reduced damage to HSCs, as evidenced by analysis of colony-forming units-granulocytes/macrophages (CFU-GM) and burst-forming units erythroid (B-FUE), especially at higher 5.8 Gy radiation doses. Gamma tocotrienol also improved circulating neutrophil and platelet recovery.

An interesting study by Gotzhein et al. [2] describes the results of experiments on various heterochronic transplantations using the most advanced technologies for cell tracking, including genetic barcoding [12,13], in combination with multicolor labeling [14]. The barcoding was performed ex vivo with various isolated classes of murine HSCs and progenitors using lentiviral transduction, followed by transplantation into lethally irradiated animals. The obtained data indicate that reconstitution was mainly driven by HSCs and multipotent progenitors (MPPs), but not the committed myeloid or lymphoid progenitors. Moreover, the authors observed that the dynamics of reconstitution and the contribution of the transduced HSPC subpopulations were largely independent of age. These results are somewhat at odds with the accepted notion, derived from the clinical experience with numerous hematopoietic stem cell transplantations, which indicates that cells from young donors (below 30 years) contribute to the better survival of recipients [15,16]. The authors also did not find the earlier-reported prominent myeloid skewing with the transplantation of aged cells [17,18,19]. The authors attribute this to ex vivo culturing and the transduction of hematopoietic cells with lentiviral barcoding constructs, which might largely eliminate the skewing effect. It should be mentioned that this explanation has quite a valid raison d’être; recent works have demonstrated the previously overlooked deleterious effect of HSC manipulations under normoxia conditions that might induce HCS activation, thus resulting in their exit from a quiescent state and their differentiation [20]. Therefore, extended ex vivo manipulations of HSCPs may introduce bias in the experimental results.

The review article by Xu et al. [3] addresses the important issue of effects of inflammatory or infectious signals on normal HSC fate, lineage output and function. The authors provide a brief analysis of how recent technological advances change our view of global hematopoiesis organization as a highly hierarchical process, emphasizing heterogeneity within the hematopoietic stem/progenitor compartment. This is followed by a succinct description of the role of the BM niche in the support and control of hematopoiesis. The authors then devote a major part of their analysis to perturbations of hematopoiesis in chronic inflammatory disease, and diabetes mellitus in particular. A large body of evidence demonstrates that hyperglycemia promotes myelopoiesis and the inflammatory macrophage phenotype. Importantly, it also induces a form of persistent “memory” in BM progenitors, which results in the continuing inflammatory phenotype supporting the chronic character of diabetes. Hyperglycemia also induces profound changes in the BM niche, resulting in the dysfunction of the endothelial component and sympathetic nervous system, as well as the elevation of inflammatory cytokine production by niche cells. Overall, these studies demonstrate the existence of a “vicious circle” of diabetes-driven low-grade inflammation that results in a further pro-inflammatory shift in both hematopoietic progenitors and the BM niche. Obesity is another example of a chronic disease with low-grade inflammation characterized by, as far as concerns hematopoiesis, loss of BM integrity, disruption of normal hematopoiesis, increased pools of myeloid progenitors and general myelopoietic bias. In addition to the effects on hematopoiesis itself, obesity also impacts the niche, substantially increasing the number of adipocytes in BM. The authors further discuss the hematopoietic alterations in trained immunity [21], a long-lasting form of innate immunological memory that enables the heightened response to the secondary challenge. The persistence of trained immunity is enabled by changes in the metabolic and epigenetic profiles in HSPCs [22], leading to enhanced myelopoiesis. In the wide group of trained immunity triggers, a prominent place belongs to β-glucans, LPS and high-fat diet.

The review article by Belyavsky et al. [4] discusses, in some detail, numerous aspects of hematopoiesis, HSPCs and mesenchymal stem/progenitor cells (MSCs). It starts with description of the embryonic hematopoiesis, and in particular, two waves of hematopoiesis in the yolk sac followed by the formation of HSCs in the AGM (aorta–gonad–mesonephros) region from the hemogenic endothelium. In addition, the most important transcription factors regulating this process are reviewed in this section. The formation of MSCs in embryo is also discussed. The authors then describe adult HSCs and the main findings of recent years concerning adult steady-state hematopoiesis, and in particular, its clonal structure as revealed by various approaches. The authors also review new perspectives on the hematopoietic hierarchy based on the latest results obtained from single-cell transcriptome analysis. In the next section of the review, a role for MSCs in adult hematopoiesis is analyzed—in particular, the differentiation hierarchy of the mesenchymal component of BM, also revealed by single-cell transcriptome analysis. The next section of the review is devoted to the aging hematopoietic system, and in particular, to the changes in HSCs that gradually lose their self-renewal and regeneration capacity, albeit apparently increasing their numbers. The review further describes changes in the mesenchymal component of BM during aging, in particular, the increased secretion of pro-inflammatory cytokines and the role of epigenetic factors, especially TET proteins and DNA methyltransferases, in MSC aging. The authors continue with a discussion of the mechanisms of HSC aging, namely, oxidative stress, DNA damage and mitochondrial malfunction, as well as defects in proteostasis mechanisms including the proteasome system, the unfolded protein control system and autophagy. The deregulation of epigenetic mechanisms and polarity loss in aged HSCs is also analyzed. In the final section of the review, approaches to the rejuvenation of the hematopoietic system and perspectives on translation to the clinic are analyzed in some detail.

The review by Kandarakov et al. [5] considers the important topic of niches of HSPCs in bone marrow. The authors first discuss the anatomy of the BM niche, including anatomy of BM in general and the location of the niche in hypoxic regions of BM. The review then considers different and often-discordant views on the nature of the BM niche for HSCs and suggests that HSCs may be associated with a number of anatomical locations; thus, niches are likely to be abundant in BM. The authors continue with a detailed analysis of potential niche cellular components and their functions, including MSCs, endothelium, osteoblasts, megakaryocytes, macrophages, adipocytes, lymphoid cells and nerve fibers, concluding this chapter with the results of a single-cell analysis of niche heterogeneity. The review then considers the niches of committed cells, which are apparently different from those of HSCs. The next section is devoted to the analysis of HSC metabolism states and their relation to the niche. The following section describes the age-related changes in the HSCs and the niche, including its specific cellular constituents. The two concluding chapters deal with niche transformation in leukemia, with its effects on leukemogenesis, and finally, with the contemporary state of niche modeling.

The review article by Shevyrev et al. [6] is focused on the complex and highly intricate issue of the origin and development of hematopoietic cells in embryo, embryonic HSCs, the development of adult HSCs and changes in hematopoiesis occurring during aging. The review starts with the early stages of HSC formation in embryo via a characterization of hemogenic endothelium and a description of events resulting in the appearance of the first HSCs precursors in the dorsal aorta. The authors continue with a characterization of the first definitive HSCs that are able to give rise to all hematopoietic lineages, stressing in particular their high regenerative potential, both in mice and in humans. The review then discusses the migration of these cells through the vascular labyrinths of the placenta to the fetal liver, where their numbers expand significantly. The authors also describe the main differences between embryonic and adult HSCs. Following this, the authors proceed to briefly characterize adult hematopoiesis, while most of the subsequent text is devoted to alterations in HSCs and hematopoiesis during aging. The review provides a comprehensive analysis of the negative processes accompanying HSC aging, including the increase in DNA damage and its underlying mechanisms, epigenetic modifications, transcriptomic alterations and the increase in cell heterogeneity. The authors discuss, in some detail, the phenomenon of clonal hematopoiesis, its mechanisms and the major genes driving it, as well as myeloid bias occurring both in clonal hematopoiesis and in normal hematopoiesis during aging. Substantial attention is also paid to inflammation and alterations in the aging BM niche. In the final part of their review, the authors describe the numerous negative effects of aging on cell composition and the operation of the immune system.

In conclusion, a broad range of topics pertaining to hematopoiesis was presented in this Special Issue through original state-of-the-art experimental articles and comprehensive and highly informative reviews. We hope that these contributions will be of substantial interest both to professionals working in this field and to the biomedical audience in general.
